# Core Mental Health Clinician Capacity and Use Rates in the US Military

**DOI:** 10.1001/jamanetworkopen.2024.34246

**Published:** 2024-09-18

**Authors:** Yu-Chu Shen, Jennifer Heissel, Marigee Bacolod

**Affiliations:** 1 Department of Defense Management, Naval Postgraduate School, Monterey, California; 2 National Bureau of Economic Research, Cambridge, Massachusetts; 3 RAND Corporation, Santa Monica, California; 4 IZA Institute of Labor Economics, Bonn, Germany

## Abstract

**Importance:**

Active duty service members have higher mental health stress and cannot choose where to live. It is imperative to understand how geographic access may be associated with their ability to obtain mental health treatment and how the COVID-19 pandemic was associated with these patterns.

**Objective:**

To identify changes in the prevalence and intensity of mental health care use when service members experienced changes in core mental health clinician (defined to include psychiatrists, psychiatric nurse practitioners, clinical psychologists and social workers, and marriage and family therapists) capacity in their communities and whether patterns changed from before to after the onset of the COVID-19 pandemic.

**Design, Setting, and Participants:**

This retrospective cohort study of the active duty population between January 1, 2016, and December 31, 2022, was conducted using individual fixed-effects models to estimate changes in the probability of mental health care visits and visit volume when a person moved across communities with adequate core mental health clinician capacity (≥1 clinician/6000 beneficiaries), shortage areas (<1 clinician/6000 beneficiaries), and areas with 0 clinicians within a 30-minute drive time. All US active duty service members stationed in the continental US, Hawaii, and Alaska were included. Data were analyzed from January through July 2024.

**Main Outcomes and Measures:**

The first set of outcomes captured the probability of making at least 1 mental health care visit in a given quarter; the second set of outcomes captured the intensity of visits (ie, the number of visits log transformed).

**Results:**

This study included 33 039 840 quarterly observations representing 2 461 911 unique active duty service members from the Army, Navy, Marines, and Air Force (1 959 110 observations among Asian or Pacific Islander [5.9%], 5 309 276 observations among Black [16.1%], 5 287 168 observations among Hispanic [16.0%], and 18 739 827 observations among White [56.7%] individuals; 27 473 563 observations among males [83.2%]; mean [SD] age, 28.20 [7.78] years). When an active duty service member moved from a community with adequate capacity at military treatment facilities to one with 0 clinicians within a 30-minute drive, the probability of a mental health visit to any clinician decreased by 1.13 percentage points (95% CI, −1.21 to −1.05 percentage points; equivalent to a 11.6% relative decrease) and the intensity of total visits was reduced by 7.7% (95% CI, −9.0% to −6.5%). The gap increased from before to after the onset of the COVID-19 pandemic, from 8.5% (equivalent to −0.82 percentage points [95% CI, −0.92 to −0.73 percentage points]) to 16.2% (equivalent to −1.58 percentage points [95% CI, −1.70 to −1.46 percentage points]) in the probability of visiting any clinician type for mental health.

**Conclusions and Relevance:**

In this study, active duty personnel assigned to locations without core military mental health clinicians within a 30-minute drive time were less likely to obtain mental health care and had fewer mental health care visits than those in communities with adequate military mental health capacity even if there was adequate coverage from the civilian sector. The care disparity increased after the onset of the COVID-19 pandemic.

## Key Points

**Question:**

Are core mental health clinician (psychiatrist, psychiatric nurse practitioner, clinical psychologist and social worker, and marriage and family therapist) shortages associated with use and intensity of mental health care among US active duty service members?

**Findings:**

In this cohort study of 2 461 911 active duty service members, those in communities with no core mental health clinicians at military treatment facilities within a 30-minute drive were statistically significantly less likely to have any treatment and had lower treatment intensity compared with those in communities with adequate core mental health clinician capacity. The gap increased from before to after the onset of the COVID-19 pandemic.

**Meaning:**

This study found that military personnel without access to core military mental health clinicians had low use of mental health care, even when there was adequate coverage among civilian core mental health clinicians nearby.

## Teaser

This cohort study investigates the association of mental health clinician shortages with prevalence and intensity of mental health use among active duty service members.

## Introduction

After the onset of the COVID-19 pandemic, there has been a surge in demand for mental health care across demographic groups, straining clinician capacity.^1^ US active duty service members experienced unique challenges in seeking care from the Military Health System (MHS). The active duty population includes enlisted service members and officers from all branches of service and traditionally has higher mental health care needs than the civilian population. MHS directly provides health services to the active duty population and other military-connected individuals at its military treatment facilities (MTFs) and purchases care from civilian clinicians and hospitals. Concerned with increasing costs of care, the Department of Defense had hoped to increase reliance on civilian clinicians as it reformed the MHS under the National Defense Authorization Act of 2017.^2^ However, a 2017 to 2020 survey of civilian clinicians found that only 33% to 37% of behavioral and mental health clinicians accepted TRICARE, the MHS health insurance program.^3,4,5^ Meanwhile, internal Department of Defense reports found that MHS failed to consistently meet access-to-care standards (as measured by different metrics of appointment wait times) for mental health services and identified shortages of mental health clinicians at MTFs.^6^

Past studies have examined financial barriers to access for mental health care and how individual traits are associated with care-seeking behavior for mental health. However, few studies to our knowledge have focused on how geographic access, in particular clinician capacity within a reasonable drive time, is associated with care-seeking behavior among the active duty population. An exception is our prior study^7^ that examined this question using data prior to the COVID-19 pandemic and examined only psychiatrist capacity. While psychiatrists are critical providers of mental health care, they represent a small fraction of overall mental health clinicians (between 11% and 15% based on tabulation of the National Provider Identifier registry^8^). Understanding how the association between a broader set of mental health clinicians capacity and care use changed from before to after the onset of the COVID-19 pandemic is critical as MHS continues its reforms and addresses resource allocations in the postpandemic era to improve mental health care for the military.^9^

In this study, we address the following question: Are core mental health clinician shortages at MTFs associated with the probability of mental health care use and the intensity of care among the US active duty population in military and civilian care settings? Using the definition from the Health Resources and Services Administration (HRSA), we defined core mental health clinicians as including psychiatrists, psychiatric nurse practitioners, clinical psychologists and social workers, and marriage and family therapists.^10^ Using longitudinal data that followed up all active duty military personnel between 2016 and 2022, we examined whether the pattern of mental health care use associated with capacity changed from before to after the onset of the COVID-19 pandemic. We hypothesized that the pandemic was associated with an exacerbated disparity in getting mental health care for members residing in communities with adequate clinicians at MTFs compared with those in communities without core mental health clinicians nearby. We focused on military mental health clinician shortages in this analysis because almost all active duty personnel reside in communities that would be classified as having adequate civilian mental health capacity (more details are given subsequently). However, our outcomes examined care that occurred in military and civilian settings, and our analysis is highly relevant to the civilian health care market, a critical component of the military health system.

## Methods

This cohort study was approved by the Naval Postgraduate School Institutional Review Board. The requirement to collect consent has been waived in accordance with 32 CFR §219.116. The study followed the Strengthening the Reporting of Observational Studies in Epidemiology (STROBE) reporting guideline.

### Study Population and Data

Our study population represented 100% of the active duty population that ever served in the US Army, Navy, Air Force, or Marine Corps from January 1, 2016, to December 31, 2022. The analytical sample included 33 039 840 year-quarter observations representing 2 461 911 unique persons.

The Defense Enrollment Eligibility Reporting System, an administrative database, provided quarterly individual demographics (sex, self-identified race and ethnicity, age, marital status, and number of dependents), service characteristics (rank, branch, reserve or active duty status, and military occupation specialty), and geographic location (mailing zip code and assigned and attached unit). The Defense Enrollment Eligibility Reporting System classified race and ethnicity into the following groups: American Indian or Alaskan Native, Asian or Pacific Islander, Hispanic, non-Hispanic Black, non-Hispanic White, other, and unknown. In this study, American Indian or Alaskan Native was combined with other race due to a low population number. We report race and ethnicity information to show that our study population reflects the general active duty population demographic distribution. The Defense Health Agency provided encounter date, diagnostic codes, and clinician characteristics for all TRICARE-paid inpatient and outpatient visits occurring in MTF or civilian settings. We identified MTF core mental health clinicians (including psychiatrists, psychiatric nurse practitioners, clinical psychologists and social workers, and marriage and family therapists per HRSA definition) capacity using the Medical Expense and Performance Reporting System and the Defense Medical Human Resource System internet. Likewise, we identified civilian core mental health clinician capacity using the National Plan and Provider Enumeration System National Provider Identifier. We used a web-based query^11^ to geocode a database of driving time between centers of each service member’s community and the MTFs and zip code centers of civilian clinicians’ practicing locations. Finally, we used data from the Federal Office of Rural Health Policy) to identify rural communities.^12^

### Defining Outcomes

A service member was identified as having a mental health visit if a medical visit contained diagnostic codes F01 to F99 from the *International Statistical Classification of Diseases, Tenth Revision, Clinical Modification* (*ICD-10-CM*).^13^ Our first dependent variable took on the value of 1 if the service member had at least 1 mental health visit in a given quarter and 0 otherwise. Our second outcome examined the intensity of visits, measured by total number of visits to each setting in a given quarter, log transformed. For both outcomes, we examined visits that occurred in MTFs, civilian settings, and any setting.

### Defining Core Mental Health Clinician Capacity

We analyzed the capacity of core mental health clinicians within a 30-minute drive of a service member’s community. HRSA defines an area as having a shortage of mental health clinicians if the relevant population to core mental health clinicians ratio exceeds 6000 to 1 for populations with unusually high mental health care needs and 9000 to 1 for populations with reference level needs.^10^ Given the nature of military life, we used 6000 to 1 in our main analysis but also report results using 9000 to 1. Using previously developed metrics^14^ (eAppendix 1 in Supplement 1), we constructed military core mental health clinician capacity as the number of core mental health clinicians per the HRSA definition working in an MTF within a 30-minute drive divided by the total number of TRICARE beneficiaries within 30 minutes of a service member’s community given that non-TRICARE beneficiaries are not eligible to use MTF services. We classified each community into 3 categories: adequate (≥1 clinician/6000 beneficiaries), shortage (<1 clinician/6000 beneficiaries), and no clinician (0 clinicians within a 30-minute drive) areas.

We created similar variables for civilian capacity, where the relevant population was all residents based on the US Census within 30 minutes of a given community and the clinician universe includes all civilian core mental health clinicians whose practice location is within 30 minutes. Virtually all active duty personnel were located in communities with adequate civilian clinicians, with 220 573 observations (0.7%) among service members with no civilian access and 1 450 192 observations (4.4%) among service members with a shortage within a 30-minute drive. Given limited variation on the civilian measure, we focused on MTF core mental health clinician capacity as the key independent variable.

### Statistical Analysis

We examined 2 sets of outcomes. First, we examined a binary outcome of whether the individual made any visit in that quarter. We implemented a linear probability model (see eAppendix 2 in Supplement 1 for full specification). The key independent variable was the 3-level categorical MTF capacity measure described previously. Critically, the linear probability model includes individual fixed effects, which captured any time-invariant unobservable characteristics of individuals, including an individual’s underlying mental health need and care-seeking preference. Under this empirical framework, changes in outcomes can be attributed to 2 sources: when a service member moved to a new location with a different capacity and when a service member experienced changes in capacity within the same location over time. Owing to the large number of fixed effects, logit models would not be appropriate because of incidental parameter problems.^15^

In our context, service members were effectively randomly assigned to a location once we conditioned on service branch and occupation.^16^ Individual fixed effects accounted for branch and occupation, meaning we removed statistical bias from selection into a location that would be present where individuals may choose to move to a location due to mental health care needs.^17^ We also included time-varying events in the model, including whether a person was demoted, was promoted to a more senior rank, transferred to a different occupation, got divorced, got married, gained a dependent child, moved to a different location, moved to a rural community, or returned from an overseas deployment in the prior or current quarter, along with year-quarter dummies to account for macro trends over time. We did not include time-invariant variables (eg, race and ethnicity) because individual fixed effects capture these characteristics. We clustered heteroskedasticity-robust standard errors within individuals to account for intraperson correlation across time.^17,18^ Overall, this model captured mean changes in usage within individuals as they experienced different capacity levels.

The second set of outcomes was the log-transformed number of visits in MTFs, civilian settings, and any setting, limited to individuals who had made at least 1 mental health visit in a given quarter. We used an ordinary least square regression with individual fixed effects, where the model specification was identical to the previously described analysis.

In our final models, we defined the period after the onset of the COVID-19 pandemic as the start of the second quarter of 2020 onward, then estimated separate coefficients for MTF capacity measures for before and after the onset of the COVID-19 pandemic. We deemed our results statistically significant at *P* < .05 using 2-sided tests. We estimated all models using Stata statistical software version 17 (StataCorp).^19^ Data were analyzed from January through July 2024.

## Results

This study included 33 039 840 quarterly observations representing 2 461 911 unique active duty service members from the Army, Navy, Marines, and Air Force (1 959 110 observations among Asian or Pacific Islander [5.9%], 5 309 276 observations among Black [16.1%], 5 287 168 observations among Hispanic [16.0%], and 18 739 827 observations among White [56.7%] individuals; 5 566 277 observations among females [16.8%] and 27 473 563 observations among males [83.2%]; mean [SD] age, 28.20 [7.78] years). Among our sample, 15 906 815 observations (48.1%) were among service members located in communities with adequate MTF core clinicians, 14 898 470 observations (45.1%) were among service members in in communities with a shortage, and the remaining 2 234 555 observations (6.8%) were among service members with no access to MTF core mental health clinicians within a 30-minute drive. When expanding the active duty population to include National Guard, Coast Guard, and activated reservists, the number with no access increased to 5 998 465 of 39 296 240 observations (15.2%). When expanding the population to all TRICARE beneficiaries, 120 979 936 of 220 190 112 observations (54.9%) among non–active duty beneficiaries (eg, family members and retirees) would be in communities without any MTF mental health clinicians (eFigure in Supplement 1). The Table shows summary statistics of the study population by MTF capacity within a 30-minute drive. The Table shows that individual characteristics were similar across the 3 types of communities and reflect the general distribution of the US active duty population, with the exception of service distribution. Army personnel were more represented in communities with adequate capacity than in communities with shortages (8 215 327 observations [51.6%] vs 3 684 233 observations [24.7%]), whereas there were relatively more Navy personnel in communities with no MTF mental health clinicians (2 405 362 observations [15.1%] in adequate communities vs 1 100 628 observations [49.3%] in no clinician communities). Individual fixed effects controlled for these service differences. The distribution of time-invariant characteristics (eg, sex and race and ethnicity) remained the same when we aggregated observations from person-quarter level to person level (eTable 1 in Supplement 1).

**Table.** Summary Statistics of Study Population

Characteristic	Person-quarters, No. (%)		
	Adequate MTF core clinicians (n = 15 906 815 [48%])^a^	Shortage (n = 14 898 470 [45%])^a^	No MTF core clinician (n = 2 234 555 [7%])^a^
Demographics			
Sex			
Female	2 737 574 (17.2)	2 465 389 (16.5)	363 314 (16.3)
Male	1 3169 241 (82.8)	1 2433 081 (83.5)	1 871 241 (83.7)
Race and ethnicity			
Asian or Pacific Islander	949 835 (6.0)	871 143 (5.8)	138 132 (6.2)
Black	2 702 504 (17.0)	2 263 950 (15.2)	342 822 (15.3)
Non-White Hispanic	2 501 846 (15.7)	2 427 233 (16.3)	358 089 (16.0)
White	9 050 864 (56.9)	8 444 602 (56.7)	1 244 361 (55.7)
Unknown or other race^b^	701 766 (4.4)	891 542 (6.0)	151 151 (6.8)
Married	8 653 869 (54.4)	7 955 954 (53.4)	1 297 061 (58.0)
Age, mean (SD), y	28.22 (7.77)	28.09 (7.78)	28.73 (8.10)
Service characteristics			
Branch			
Army	8 215 327 (51.6)	3 684 233 (24.7)	559 142 (25.0)
Navy	2 405 362 (15.1)	4 582 439 (30.8)	1 100 628 (49.3)
Air Force	3 723 964 (23.4)	3 830 777 (25.7)	334 418 (15.0)
Marines	1 562 162 (9.8)	2 801 021 (18.8)	240 367 (10.8)
Enlisted	1 3200 000 (83.0)	12 200 000 (81.9)	1 869 916 (83.7)
Officer	2 714 772 (17.1)	2 665 490 (17.9)	364 426 (16.3)
Time-varying characteristics			
Got divorced	150 565 (0.9)	137 195 (0.9)	30 199 (1.4)
Got married	267 752 (1.7)	258 572 (1.7)	32 545 (1.5)
Gained a nonspouse dependent	408 066 (2.6)	365 189 (2.5)	49 108 (2.2)
Promoted	439 121 (2.8)	424 755 (2.9)	41 449 (1.9)
Demoted	66 994 (0.4)	60 851 (0.4)	6481 (0.3)
Transferred to a different occupation	284 649 (1.8)	435 214 (2.9)	55 883 (2.5)
Moved to a different community	4 104 409 (25.8)	3 806 467 (25.5)	755 684 (33.8)
Returned to US in prior quarter	135 748 (0.9)	130 207 (0.9)	15 816 (0.7)
Returned to US in current quarter	179 394 (1.1)	200 044 (1.3)	19 436 (0.9)
Geographic characteristics			
Reside in rural community	3 696 034 (23.2)	699 151 (4.7)	171 376 (7.7)
No. civilian clinicians per 10 000 population, median (IQR)	49.73 (15.82-112.73)	23.75 (11.25-48.89)	19.72 (4.82-27.71)
Mental health care visit status			
Visited MTF for mental health care	1 547 365 (9.7)	1 229 082 (8.2)	131 047 (5.9)
No. MTF visits if ≥1, mean (SD)	4.04 (5.25)	3.92 (5.26)	3.21 (4.26)
Visited civilian clinician	266 931 (1.7)	266 930 (1.8)	88 227 (3.9)
No. civilian visits if ≥1, mean (SD)	5.17 (7.56)	5.00 (7.02)	3.90 (5.53)
Mental health care visit to MTF or civilian setting	1 661 165 (10.4)	1 352 247 (9.1)	198 126 (8.9)
No. visits to any setting if ≥1, mean (SD)	4.50 (6.34)	4.47 (6.33)	3.79 (5.47)

Abbreviation: MTF, military treatment facility.

^a^ Core mental health clinician capacity was defined by the number of clinicians within a 30-minute drive time (adequate: ≥1 clinician/6000 beneficiaries; shortage: <1 clinician/6000 beneficiaries). Per the Health Resources and Services Administration, core mental health clinicians include psychiatrists, psychiatric nurse practitioners, clinical psychologists, clinical social workers, and marriage and family therapists.

^b^ Other race includes American Indian or Alaskan Native and race and ethnicity groups not identified previously.

The bottom panel of the Table shows that the share of active duty personnel who visited an MTF for mental health care was 1 547 365 observations (9.7%) for those in adequate capacity communities and 131 047 observations (5.9%) among those in a community with no mental health clinician within 30-minute drive; similarly, there was a lower intensity of visits as the availability of MTF mental health clinicians decreased. Less than 2% of personnel received mental health care in civilian settings in communities with adequate MTF mental health clinicians (266 931 observations [1.7%]); the rate was close to 4% in communities with no MTF mental health clinicians (88 227 [3.9%]). However, the intensity of visits in civilian settings was lower in such communities (mean [SD] 3.90 [5.53] visits per quarter vs 5.17 [7.56] visits per quarter in adequate communities).

**Figure 1.**
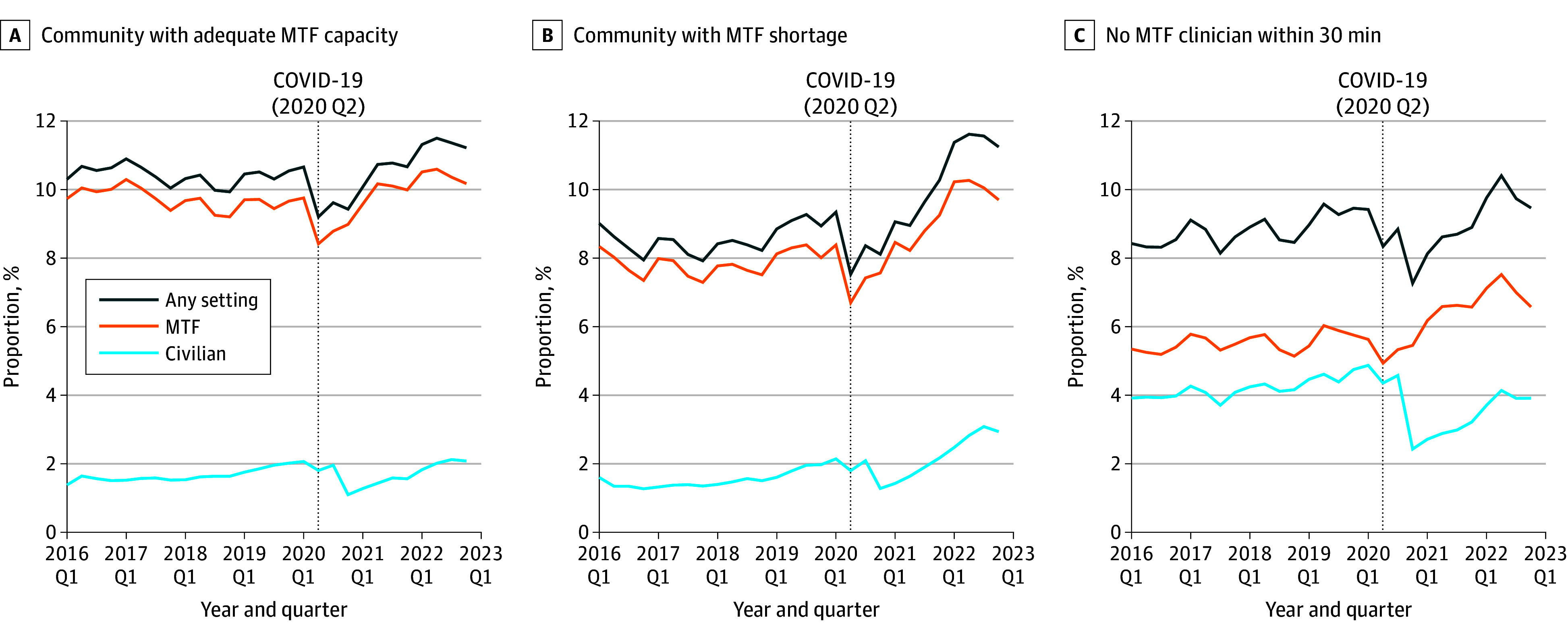
Proportion of Active Duty Service Members With ≥1 Mental Health Care Visit by Quarter Adequate capacity was defined as 1 or more core mental health clinician per 6000 beneficiaries, and shortage as less than 1 core mental health clinician per 6000 beneficiaries. MTF indicates military treatment facility.

Next, we examined the trend in mental health visits by MTF clinician capacity before and after the start of the COVID-19 pandemic. Figure 1 shows the time trend between January 2016 and December 2022 of the percentage of our study population who made at least 1 mental health visit per quarter by MTF core mental health capacity level within a 30-minute drive. First, across all communities, visit probability decreased at the start of the COVID-19 pandemic but increased steadily after the onset of the COVID-19 pandemic so that 122 129 service members (11.1%) made at least 1 mental health visit at the last quarter of 2022. Second, while there was a small upward trend in visits to civilian settings after the onset of the COVID-19 pandemic, most care still occurred in MTFs even in communities where individuals had to spend more than 1 hour on the road round trip to MTF mental health clinicians. Third, service members in communities with no MTF mental health clinician within 30 minutes consistently had a lower probability of receiving mental health care in any setting across the entire period, and they relied more on civilian clinicians. Moreover, civilian use decreased more at the start of the COVID-19 pandemic in communities with no MTF mental health clinicians (2.5 percentage point decrease, from 4030 of 82 730 observations in first quarter of 2020 [4.9%] to 2014 of 82 928 observations in fourth quarter of 2020 [2.4%]) than in the other 2 types of communities. Not only did service members in communities without mental health clinicians at MTFs within a 30-minute drive exhibit a consistently lower likelihood of accessing mental health care across all settings, but their care was also markedly more compromised during the COVID-19 pandemic.

**Figure 2.**
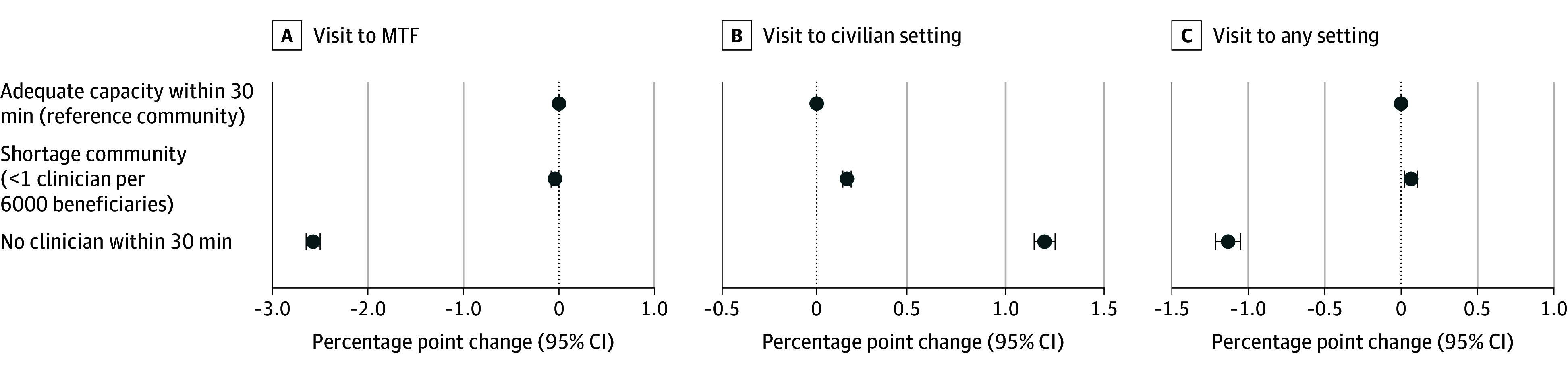
Changes in Probability of Mental Health Visit After Mental Health Capacity Changes at Military Treatment Facilities (MTFs) Changes are shown in 33 039 840 observations among service members when they experienced mental health capacity changes at MTF. The baseline mean (SD) rate of visits per quarter was 8.8% (28.3%) to MTFs, 1.7% (13.6%) to civilian settings, and 9.7% (29.6%) to any setting.

Figure 2 presents estimates from our main model on the first set of binary outcomes (see complete regression results in eTable 2 in Supplement 1). The visit probability was substantially lower when a service member moved from a community with adequate mental health capacity to one without a mental health clinician within 30 minutes or when the same community’s nearby MTF mental health capacity was reduced to zero. Specifically, the MTF visit probability was reduced by 2.57 percentage points (95% CI, −2.65 to −2.50 percentage points) when mental health capacity changed from adequate to zero within a 30-minute drive. Given the mean (SD) rate of 8.8% (28.3%), this is equivalent to a 29.2% relative decrease. This is offset by a 1.19 percentage point (95% CI, 1.13 to 1.24 percentage point; mean [SD] rate, 1.9% [13.6%]) increase in visits to civilian settings. However, because civilian visits were a small part of overall visits, the visit probability in any setting decreased by 1.13 percentage points (95% CI, −1.21 to −1.05 percentage points) or 11.6% (mean [SD] rate, 9.7% [29.6%]). Differences in visit probability were small when service members changed from adequate areas to those with a shortage in capacity and vice versa.

**Figure 3.**
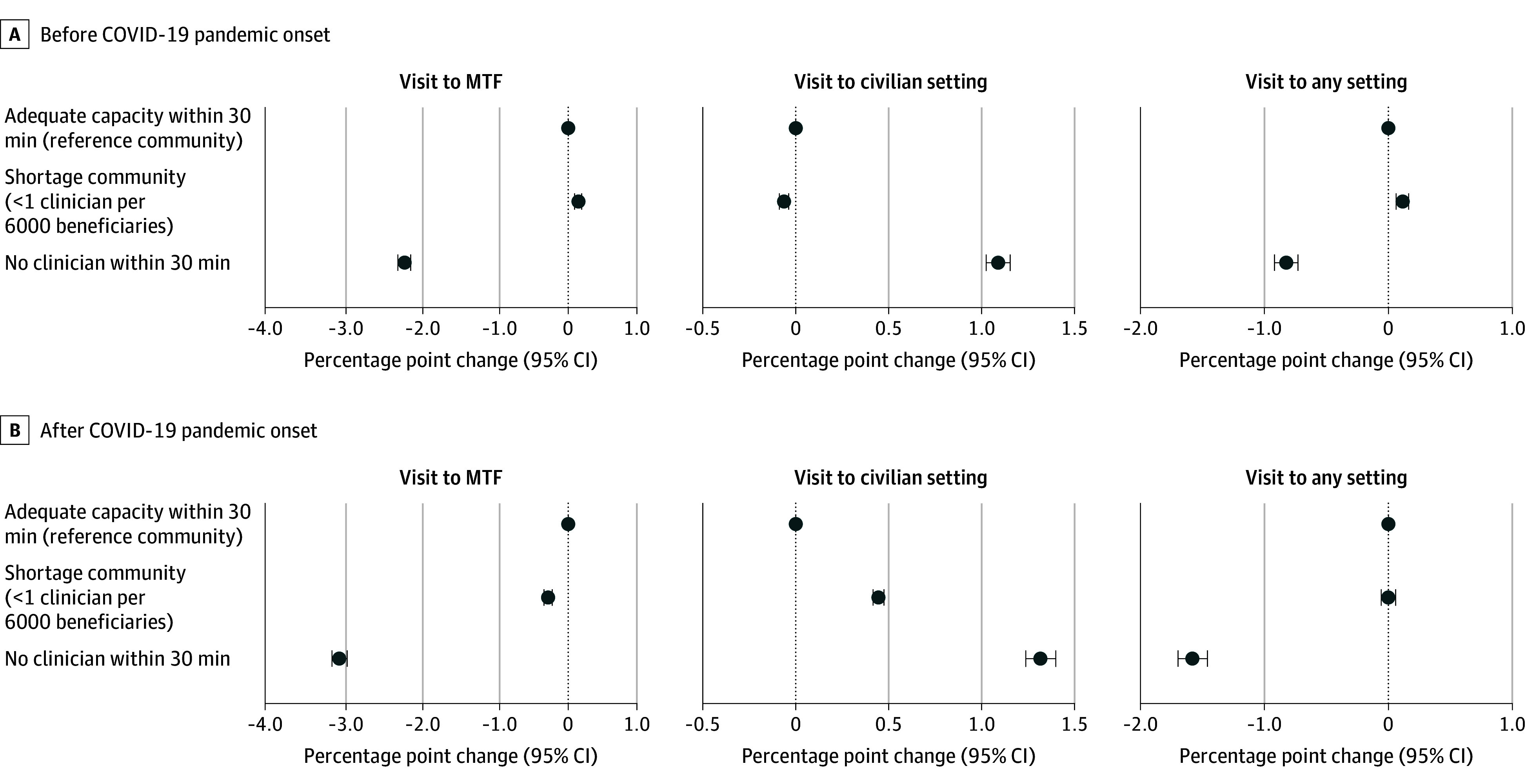
Changes in Probability of Mental Health Visit After Mental Health Capacity Changes by COVID-19 Period Changes are shown in 33 039 840 observations among service members when they experienced mental health capacity changes at military treatment facilities (MTFs). The baseline mean (SD) rate of visits per quarter was 8.8% (28.3%) to MTFs, 1.7% (13.6%) to civilian settings, and 9.7% (29.6%) to any setting. The onset of the COVID-19 pandemic is defined as the second quarter of 2020.

Figure 3 presents coefficient estimates from the set of models in which we estimated capacity coefficients separately for before and after the onset of the COVID-19 pandemic (see detailed results in eTable 3 in Supplement 1). These estimates show a larger gap in obtaining care between communities with and without adequate mental health clinicians after the onset of the COVID-19 pandemic. Specifically, MTF visit probability decreased by 3.09 percentage points (95% CI, −3.19 to −2.98 percentage points; equivalent to a 35.1% decrease given the mean [SD] rate of 8.8% [28.3%]) when moving from adequate to no clinician communities after the onset of the COVID-19 pandemic; before the COVID-19 pandemic, this disparity in visit rate was 2.21 percentage points (95% CI, −2.30 to −2.13 percentage points). The disparity in overall visit probability regardless of setting was 0.82 percentage points (95% CI, −0.92 to −0.73 percentage points; equivalent to 8.5% given the mean [SD] rate of 9.6% [29.6%]) before and 1.58 percentage points (95% CI, −1.70 to −1.46 percentage points; equivalent to 16.2%) after the onset of the COVID-19 pandemic. The before and after COVID-19 pandemic gap differences were statistically significant from one another at the .05 level.

**Figure 4.**
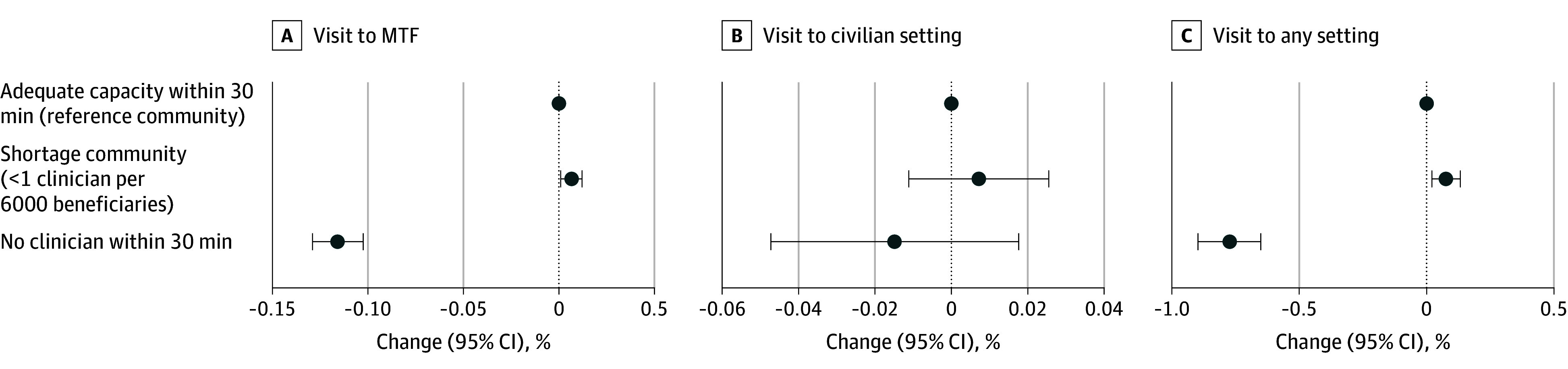
Changes in Mental Health Visit Volume After Mental Health Capacity Changes Changes are shown among service members who made at least 1 visit (2 907 494 observations for MTFs, 622 088 observations for civilian settings, and 3 211 538 observations for any setting) when they experienced mental health capacity changes at military treatment facilities (MTFs). The dependent variable is log transformed, so coefficients represent the percentage change (eg, −0.12 represents a 12% reduction).

Figure 4 presents main model estimates on intensity outcomes. Because the dependent variable is log transformed, coefficients represent percentage changes.^20^ Figure 4A shows that conditional on having at least 1 visit, MTF visit intensity decreased by 11.6% (95% CI, −12.9% to −10.2%) when a service member’s local capacity changed from adequate to no clinician, with no change in civilian visit intensity and an overall decrease of 7.7% (95% CI, −9.0% to −6.5%) in total visit volume. Visit intensity was similar for service members moving between adequate and shortage communities (see complete results in eTable 2 in Supplement 1) and similar between periods before and after the onset of the COVID-19 pandemic (eTable 3 in Supplement 1).

In our sensitivity analysis, we redefined shortage communities as those with fewer than 1 core mental health clinician per 9000 relevant population; results remained robust to this definition (eTable 4 in Supplement 1). In another sensitivity analysis, we used a high-dimensional fixed-effects model, including individual and community fixed effects to eliminate unobserved differences across communities; our results remained robust to this specification (eTable 5 in Supplement 1). We believe our main model is superior to this more restrictive specification because the more restrictive model removed valid cross-location variations in capacity.

## Discussion

This cohort study found that 7% of active duty service members lived in areas with no MTF core mental health clinicians within 30 minutes. While they had small increases in obtaining care via civilian clinicians, their overall probability of obtaining mental health care in any setting was 11.6% lower compared with service members living in communities with adequate MTF mental health clinicians, and the disparity widened over time (from 8.5% before to 16.2% after the onset of the COVID-19 pandemic).

The care disparity was also reflected in the intensity of visits. When service members moved from communities with adequate MTF mental health clinicians to those with zero clinicians available, visit volume was 11.6% lower in the MTF setting and 7.7% lower in any setting. The association between clinician capacity and intensity of care remained similar between periods before and after the onset of the COVID-19 pandemic. The persistent gap in visit intensity is important because mental health conditions typically require multiple sessions of treatment and routine follow-up. Our results showed that prevalence and intensity of visits were lower when service members moved to communities with no easy geographic access to MTF core mental health clinicians.

Disparity in care will likely be higher when looking at all TRICARE beneficiaries. While 6.8% of the active duty population from the 4 service branches were in communities with no core mental health clinicians within a 30-minute drive, the number increased to 15.2% when we included National Guard, Coast Guard, and activated reservists, and 54.9% of non–active duty dependents and retirees lived in such communities.

Our analysis also revealed a sharp decrease in use of civilian care at the start of the COVID-19 pandemic in communities with no MTF core mental health clinicians within a 30-minute drive, and the share of civilian care engagement never returned to the pre–COVID-19 pandemic level. This is in contrast to the use pattern of civilians during the same period, among whom the percentage of the adult civilian population aged 18 to 44 years with any outpatient mental health care per year increased from 12.4% in 2018 to 15.2% in 2021.^21^ This is especially concerning given that civilian care plays an even more critical role in communities lacking military clinicians. Further research would need to explore the cause of this decrease and policy levers that may prevent further erosion of civilian clinician engagements.

Past studies have highlighted nongeographic barriers as contributors to disparity in care. Our study found that geographic access was also critical; for mental health care, how easy it is to get to the clinic was associated with the probability of visits and intensity of visits. Despite a rapid increase in use of telehealth, especially in civilian mental health care after the onset of the COVID-19 pandemic,^22,23^ our study suggests that telehealth did not close the gap associated with geographic access in the military, and indeed the gap widened after the onset of the COVID-19 pandemic.

The Department of Defense might consider a 2-pronged approach to reduce disparities in care due to geographic access. One approach may involve identifying communities that have no access to a mental health clinician at an MTF within a reasonable driving time and create additional incentive pays for civilian clinicians in these areas. However, improving civilian clinician pay alone may not be enough to bring parity. Even in communities with no easy access to MTF, service members still rely on MTFs more than on civilian clinicians due to the need for referrals and other nongeographic, nonfinancial barriers to civilian clinicians. The second approach would require increased staffing and the mobility of these staff from the MTF. This would be consistent with a recent MHS call to increase the number of medical personnel to deliver health care at military facilities.^9^ In addition, the Veterans Health Administration has found some success by integrating mental health specialists in the primary care clinic for treating veterans with psychiatric illness,^24^ as well as developing a robust telehealth system.^25^ A combination of these strategies tailored for local regions, ranging from mobile clinics and satellite treatment facilities to telehealth, are all potential tools to address the geographic disparity in care.

### Limitations

This study has several limitations. Our analysis did not separately address the modality of mental health care delivery, such as the use of telehealth services, given that this was beyond the current scope of our study. However, we did capture virtual visits in our analysis, and even with the inclusion of these visits, service members with no MTF mental health clinicians nearby were less likely to receive care. Additionally, we recognize that numerous nongeographic barriers impede access for service members. By narrowing our scope to geographic access, our aim was to shed light on a critical aspect of the physical environment and how clinician capacity, which can be altered using policy levers, may be associated with use.

While we focused on the capacity of core mental health clinicians, we recognize the nuanced nature of mental health care delivery within the MHS. Notably, mental health diagnoses were often made by primary care physicians, while follow-up visits typically involved referrals to mental health specialists. Our HRSA-based measure of core mental health clinician capacity did not include primary care physicians. However, although primary care physicians played a role in diagnosing mental health conditions, our observation of a substantial disparity in visit probability and intensity when there were no core mental health clinicians in the area further underscores the importance of specialized mental health care access.

More broadly, doing a full accounting of all categories of clinicians in MHS was beyond the scope of this study. Therefore, we cannot ascertain whether communities with inadequate access to core mental health clinicians also experienced inadequate access to other health practitioners.

We also note potential measurement errors in calculating capacity, particularly in MTF data. For instance, marriage and family therapists do not have their own occupation categories and were typically classified as clinical psychologists or clinical social workers.^26^ While this may lead to underestimation of the core mental health clinician capacity within MTFs, our validation efforts indicated minimal impact on our estimations.

Additionally, our analysis focused on core mental health capacity based on the HRSA definition, which suggests that nearly all active duty service members had access to an adequate number of civilian core mental health clinicians within a 30-minute drive. However, only 33% to 37% of behavioral clinicians accept TRICARE patients, highlighting persistent challenges faced by service members in accessing mental health care in civilian settings.^3,4,5^

## Conclusions

In this cohort study of 2 461 911 active duty service members between 2016 and 2022, service members residing in communities with no core mental health clinicians at MTFs within a 30-minute drive consistently experienced a lower likelihood of having any mental health visit and lower intensity of visits compared with those in communities with an adequate number of clinicians. The gap in probability of any mental health visit between places with and without adequate capacity increased from 8.5% before to 16.2% after the onset of the COVID-19 pandemic. The lack of MTF mental health capacity has implications for dependents of active duty service members and retirees because the most non–active duty beneficiaries reside in such communities.

## Notes

Supplement 1. **eAppendix 1.** Technical supplement on data and methods to define relevant geographic access area from patient perspective **eAppendix 2.** Technical supplement on statistical model details **eFigure.** Overall TRICARE beneficiary distribution by core MTF mental health clinician capacity, separately for active duty and non–active duty population **eTable 1.** Summary statistics of the study population, reported at the person level based on last quarter of observation. **eTable 2.** Complete results of main models **eTable 3.** Separate coefficient estimates by COVID-19 period **eTable 4.** Sensitivity analysis using alternate threshold to define clinician shortage (<1 clinician per 9000 relevant population) **eTable 5.** Sensitivity analysis using 2-dimensional fixed-effects models (individual and community fixed effects)

Supplement 2.
Data Sharing Statement
