# Supplementary material for: Core Mental Health Clinician Capacity and Use Rates in the US Military
Source: JAMA Netw Open. 2024 Sep 18;7(9):e2434246. doi: 10.1001/jamanetworkopen.2024.34246 (PMC11411380; doi:10.1001/jamanetworkopen.2024.34246)
Supplement: Supplement 1. — eAppendix 1. Technical supplement on data and methods to define relevant geographic access area from patient perspective eAppendix 2. Technical supplement on statistical model details eFigure. Overall TRICARE beneficiary distribution by core MTF mental health clinician capacity, separately for active duty and non–active duty population eTable 1. Summary statistics of the study population, reported at the person level based on last quarter of observation. eTable 2. Complete results of main models eTable 3. Separate coefficient estimates by COVID-19 period eTable 4. Sensitivity analysis using alternate threshold to define clinician shortage (<1 clinician per 9000 relevant population) eTable 5. Sensitivity analysis using 2-dimensional fixed-effects models (individual and community fixed effects) [file jamanetwopen-e2434246-s001.pdf]

Supplemental Online Content

Shen Y, Heissel J, Bacolod M. Core mental health clinician capacity and use rates in the US military. *JAMA Netw Open*. 2024;7(9):e2434246.
doi:10.1001/jamanetworkopen.2024.34246

eAppendix 1. Technical supplement on data and methods to define relevant geographic access area from patient perspective

eAppendix 2. Technical supplement on statistical model details

eFigure. Overall TRICARE beneficiary distribution by core MTF mental health clinician capacity, separately for active duty and non–active duty population

eTable 1. Summary statistics of the study population, reported at the person level based on last quarter of observation.

eTable 2. Complete results of main models

eTable 3. Separate coefficient estimates by COVID-19 period

eTable 4. Sensitivity analysis using alternate threshold to define clinician shortage (<1 clinician per 9000 relevant population)

eTable 5. Sensitivity analysis using 2-dimensional fixed-effects models (individual and community fixed effects)

This supplemental material has been provided by the authors to give readers additional information about their work.

© 2024 Shen Y et al. *JAMA Network Open.*

eAppendix 1. Technical supplement on data and methods to define relevant geographic access area from patient perspective

We combine monthly data from January 2016 to December 2022 to form our analysis. We will use the term "core mental health provider" throughout the technical supplement as defined by the Health Resources and Services Administration (HRSA). Per HRSA definition, core mental health provider includes psychiatrists, psychiatric nurse practitioners, clinical psychologists, clinical social workers, marital therapists. First, we use the Defense Enrollment Eligibility Reporting System (DEERS) to capture beneficiary population size, demographic, and military branch of service characteristics of TRICARE beneficiaries for a given community. Second, we use the Medical Expense and Performance Reporting System (MEPRS) and the Defense Medical Human Resource System internet (DMHRSi) to capture MTF core mental health provider (as defined above by HRSA) capacity; and the National Plan and Provider Enumeration System National Provider Identifier (NPI) data to identify civilian core mental health providers and their practice ZIP code. Third, we use the U.S. Census, American Community Survey (ACS), and the Social Determinants of Health Database to capture the community's overall population and socioeconomic characteristics.[1] Finally, we use a web-based query[2] to derive database of driving time between centers of each ZIP code community and (1) the MTF's and (2) ZIP code centers of civilian core mental health providers' practicing location.

We use ZIP codes to define community because it is the smallest geographic unit we can capture across data sources. However, the closest core mental health provider is not necessarily practicing in the same ZIP code as the patient, especially in cities, so we need to define a more robust geographic boundary to assess patients' access to mental health providers. Rather than using the catchment area approach which defines relevant geographic boundaries from a facility's perspective, we define geographic coverage from the patient's perspective. For each

© 2024 Shen Y et al. *JAMA Network Open.*

community, we use travel time concept to define the relevant access area, rather than alternative definitions such as political boundaries (i.e., state or county lines) or fixed radius approach (such as the 20-mile radius catchment area used by the Defense Health Agency) for several reasons. First, using travel time does not impose arbitrary limits on actual access like political boundaries—an Idaho patient who lives near the state line of Oregon would have easier access to a provider in a certain part of Oregon than in Idaho. Second, unlike a fixed-mile radius approach which cannot capture geographical barriers such as mountains and water bodies, the driving time approach captures geographic access more accurately and consistently. Third, travel time is positively associated with treatment attrition. For example, female veterans are more likely to attrite from Veteran's Heath Administration care the longer their drive time, particularly for new patients[3], and longer travel time is associated with fewer annual visits for depression treatment.[4]

Researchers have used 30 minutes of travel time as a benchmark for various medical treatments such as emergency caesarean delivery,[5] cardiac care,[6,7] and opioid treatment[8]; while others have used a linear measure of time and its relationship to care.[3,4,9–11] The U.S. Department of Health and Human Services uses 30 minutes travel time as the rational area of coverage for primary care and 40 minutes for dental and mental health care.[12] We chose 30 minutes car driving time (1-hour round trip) as a reasonable time threshold that TRICARE beneficiaries are willing to travel on a repeated basis since each mental health episode can require multiple follow-up visits. It should be noted that while driving time accounts for large percent of travel time, actual travel time would be longer than the 30-minute driving time due to various factors, such as wait time for the ride, navigating parking structure, and in the case of public transportation, frequent stops.

© 2024 Shen Y et al. *JAMA Network Open.*

Based on the above geographic access definition, we take the following steps to capture the collection of ZIP codes and MTFs with core mental health providers that are within a 30-minute driving time of a given community. First, for each ZIP code where we have a TRICARE beneficiary or civilian providers, we obtain longitude and latitude coordinates of the ZIP code interior center based on the US Census. Second, for each MTF, we obtain longitude and latitude coordinates via automated web interface based on their physical address. Last, we derived a travel-time database using web-based queries that identify driving time under normal traffic conditions between each pair (ZIP code to ZIP code for care through a civilian provider and ZIP code to MTF for MTF-provided care). All core mental health providers practicing in ZIP codes and MTFs that can be reached within a 30-minute driving time from a given community would be considered geographically accessible to beneficiaries for that community.

© 2024 Shen Y et al. *JAMA Network Open.*

eAppendix 2. Technical supplement on statistical model details

We use the following model, specified as either a linear probability model (LPM) or ordinary least squares (OLS) estimate for individuals *i* in year-quarter *t* as follows:

$$Y_{it} = \beta_1 Shortage_{it} + \beta_2 NoMTFProvider_{it} + \boldsymbol{X_{it}}\delta + \tau_t + \theta_i + \varepsilon_{it}$$

where $\boldsymbol{X_{it}}$ is a vector of time-varying characteristics (whether a person was demoted, promoted to more senior rank, transferred to a different occupation, divorced, got married, gained a dependent child, moved to a different location, resided in a rural community, returned from an overseas deployment in the prior or current quarter) to account for time-varying characteristics and τ_t are year-quarter dummies to account for macro trends over time. The individual fixed effects θ_i capture any time-invariant unobservable characteristics of individuals, including an individual's underlying mental health need and care-seeking preference, race/ethnicity, and branch of service. $Shortage_{it}$ and $NoMTFProvider_{it}$ are mutually exclusive indicator variables indicating the indicating whether individual *i* lives in a shortage or no-provider location in time *t*, with the excluded category being living in a location with adequate coverage. Then, β_1 measures the effect of a change from an adequate coverage to a shortage area, after accounting for constant individual characteristics θ_i, factors that changed for everyone over time τ_t, and the individual-specific time-varying factors $\boldsymbol{X_{it}}$; while β_2 measures the effect of a change from an adequate coverage to a no-provider area. The coefficients , β_1 and β_2 are only estimated based on those who move between location types or whose community experienced capacity changes; those who are always in the same sort of location with no change in local provider capacity would have a constant value for $Shortage_{it}$ and $NoMTFProvider_{it}$ which would be captured by the fixed effect. Overall, this model captured the average changes in usage within individuals as they experience different capacity levels.

Technical supplement on results from additional analysis

Appendix eFigure. Overall TRICARE beneficiary distribution by core MTF mental health provider capacity, separately for active duty and non-active duty population

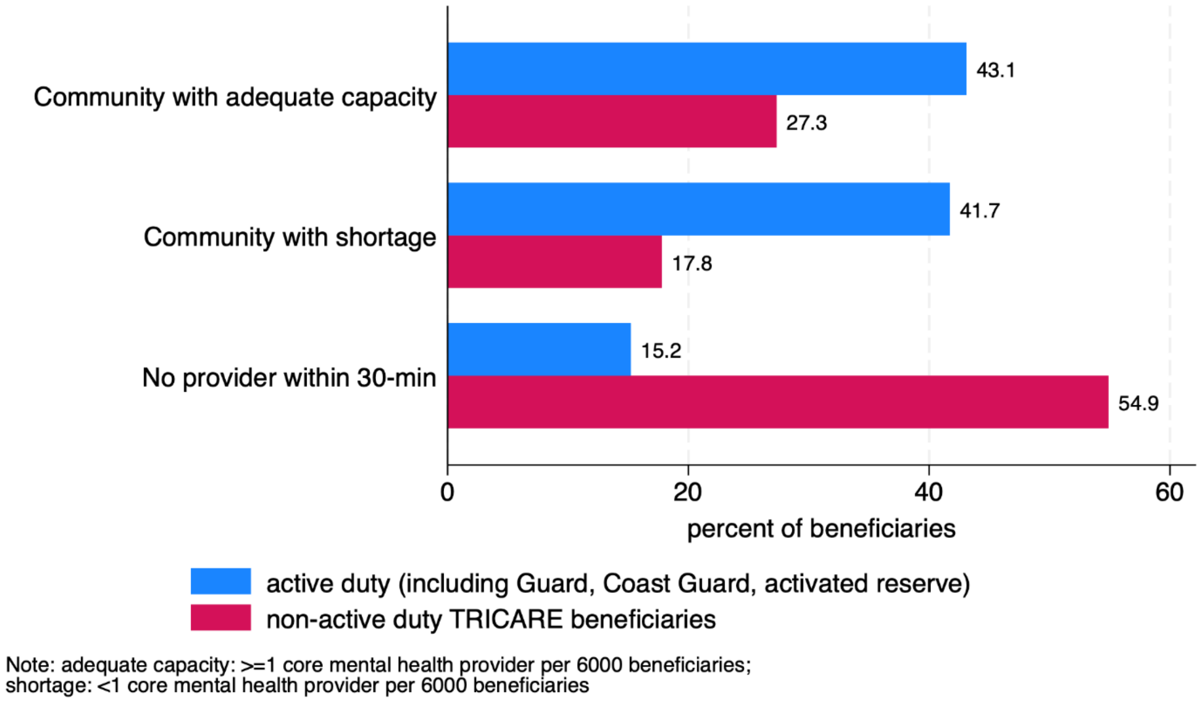

© 2024 Shen Y et al. *JAMA Network Open.*

Appendix eTable 1 Summary statistics of the study population, reported at the person level based on last quarter of observation.

	Adequate MTF core providers		Shortage (<1 provider per 6000 beneficiaries)		No MTF core provider	
No of individuals	1,076,952	44%	1,133,310	46%	251,649	10%
Service member demographics at last observed qtr						
Female	190,877	18%	192,944	17%	42,642	17%
Male	886,075	82%	940,366	83%	209,007	83%
Asian or Pacific Islander	63,535	6%	69,587	6%	14,254	6%
Black	187,213	17%	181,791	16%	38,348	15%
Non-white Hispanic	176,527	16%	199,149	18%	41,641	17%
White	605,711	56%	621,914	55%	142,389	57%
Unknown or other race	43,966	4%	60,869	5%	15,017	6%
Married	573,709	53%	590,944	52%	134,948	54%
Age (mean/SD)	28.30	8.06	28.11	8.04	28.20	8.87
Service characteristics at last observed qtr						
Army	562,492	52%	323,417	29%	53,594	21%
Navy	159,720	15%	323,706	29%	111,703	44%
Air Force	221,493	21%	271,336	24%	39,139	16%
Marines	133,247	12%	214,851	19%	47,213	19%
Enlisted	931,552	86%	970,710	86%	222,602	88%
Officer	145,389	14%	162,516	14%	29,039	12%
Geographic characteristics at last observed qtr						
Reside in rural community	248,871	23%	59,258	5%	28,164	11%
No of civilian providers per 10,000 (median/IQR)	41.26	85.6<br>7	22.65	49.5<br>6	13.02	23.7<br>1
Mental health care visit status at last observed qtr						
Visited MTF for mental health care	141,652	13%	119,196	11%	25,369	10%
No of MTF visits, if had at least 1 (mean/SD)	3.41	4.21	3.35	4.36	2.34	2.91
Visited civilian provider	25,192	2%	29,418	3%	8,662	3%
No of civilian visits, if had at least 1 (mean/SD)	4.71	5.83	4.86	5.73	4.21	5.62
Had mental health care visit	153,222	14%	134,208	12%	31,232	12%
No of visits, if had at least 1 (mean/SD)	3.88	5.03	3.99	5.32	3.04	4.34

Note: Unit of observation is an individual. Cell entries report no. of observations and percent with that characteristic, unless otherwise noted. Other race includes American Indian/Alaskan Native and race and ethnicity groups not identified above.

© 2024 Shen Y et al. *JAMA Network Open.*

Appendix eTable 2a Complete results of main model—prevalence of visits

Model specification: linear probability model with individual fixed effects and all variables listed in the table below.

	Probability of any visit		
	**MTF**	**civilian**	**Any setting**
**Military Treatment Facility core mental health provider capacity**			
adequate	0.00	0.00	0.00
	[0.00,0.00]	[0.00,0.00]	[0.00,0.00]
shortage (<1 provider per 6000 beneficiaries)	-0.04*	0.16**	0.06**
	[-0.08,-0.00]	[0.14,0.18]	[0.02,0.11]
no MTF core provider	-2.57**	1.19**	-1.13**
	[-2.65,-2.50]	[1.13,1.24]	[-1.21,-1.05]
**Individual time-varying characteristics**			
Married	0.38**	-0.01	0.37**
	[0.32,0.44]	[-0.04,0.02]	[0.31,0.43]
Got divorced	0.62**	-0.12**	0.55**
	[0.50,0.73]	[-0.18,-0.06]	[0.44,0.67]
Got married	0.01	-0.07**	-0.01
	[-0.06,0.08]	[-0.10,-0.04]	[-0.08,0.06]
Demoted	16.15**	1.75**	16.07**
	[15.92,16.39]	[1.64,1.87]	[15.84,16.30]
Added a dependent	-0.39**	-0.10**	-0.41**
	[-0.44,-0.33]	[-0.13,-0.07]	[-0.46,-0.35]
Promoted to senior rank	-0.23**	-0.11**	-0.27**
	[-0.28,-0.18]	[-0.13,-0.09]	[-0.32,-0.22]
Transferred to a different occupation category	-0.16**	-0.04**	-0.12**
	[-0.21,-0.11]	[-0.06,-0.01]	[-0.17,-0.07]
Moved to different community	-1.28**	-0.31**	-1.45**
	[-1.31,-1.26]	[-0.33,-0.30]	[-1.47,-1.42]
Returned to US in prior quarter	-0.19**	-0.23**	-0.37**
	[-0.29,-0.09]	[-0.28,-0.18]	[-0.47,-0.26]
Return to US in current quarter	-3.09**	-0.89**	-3.52**
	[-3.16,-3.02]	[-0.92,-0.86]	[-3.59,-3.45]
Reside in rural community	-0.03	0.13**	0.02
	[-0.09,0.03]	[0.11,0.16]	[-0.04,0.09]
number of civilian providers per capita (log transformed)	-0.16**	-0.60**	-0.49**
	[-0.23,-0.10]	[-0.63,-0.56]	[-0.56,-0.43]

© 2024 Shen Y et al. *JAMA Network Open.*

quadratic of number of civilian providers per capita (log transformed)	0.09**	0.17**	0.17**
	[0.08,0.10]	[0.16,0.17]	[0.16,0.19]
**Year-quarter indicators**			
2016 Q1	0.00	0.00	0.00
	[0.00,0.00]	[0.00,0.00]	[0.00,0.00]
2016 Q2	0.35**	0.08**	0.39**
	[0.30,0.40]	[0.05,0.11]	[0.34,0.44]
2016 Q3	0.79**	0.19**	0.90**
	[0.73,0.84]	[0.16,0.22]	[0.84,0.96]
2016 Q4	1.23**	0.25**	1.37**
	[1.17,1.29]	[0.22,0.28]	[1.30,1.43]
2017 Q1	2.20**	0.42**	2.37**
	[2.14,2.26]	[0.38,0.45]	[2.31,2.44]
2017 Q2	2.55**	0.57**	2.76**
	[2.48,2.61]	[0.53,0.60]	[2.70,2.83]
2017 Q3	2.63**	0.67**	2.90**
	[2.57,2.70]	[0.64,0.70]	[2.83,2.97]
2017 Q4	2.94**	0.76**	3.27**
	[2.87,3.00]	[0.73,0.80]	[3.20,3.34]
2018 Q1	3.78**	0.90**	4.15**
	[3.71,3.85]	[0.87,0.94]	[4.08,4.22]
2018 Q2	4.22**	1.07**	4.67**
	[4.15,4.29]	[1.04,1.10]	[4.60,4.74]
2018 Q3	4.36**	1.23**	4.90**
	[4.29,4.43]	[1.20,1.27]	[4.83,4.97]
2018 Q4	4.71**	1.30**	5.27**
	[4.64,4.78]	[1.27,1.34]	[5.20,5.35]
2019 Q1	5.55**	1.49**	6.17**
	[5.47,5.62]	[1.46,1.53]	[6.09,6.24]
2019 Q2	5.94**	1.70**	6.64**
	[5.87,6.01]	[1.66,1.74]	[6.57,6.72]
2019 Q3	6.26**	1.94**	7.09**
	[6.19,6.33]	[1.90,1.98]	[7.01,7.16]
2019 Q4	6.64**	2.09**	7.55**
	[6.57,6.72]	[2.06,2.13]	[7.47,7.63]
2020 Q1	7.18**	2.28**	8.15**
	[7.11,7.25]	[2.24,2.32]	[8.07,8.23]
2020 Q2	5.93**	2.01**	6.77**
	[5.86,6.00]	[1.97,2.04]	[6.69,6.85]

© 2024 Shen Y et al. *JAMA Network Open.*

2020 Q3	6.95**	2.38**	7.94**
	[6.88,7.03]	[2.34,2.41]	[7.86,8.02]
2020 Q4	7.45**	1.54**	7.99**
	[7.38,7.53]	[1.50,1.57]	[7.92,8.07]
2021 Q1	8.46**	1.81**	9.11**
	[8.39,8.54]	[1.77,1.84]	[9.03,9.19]
2021 Q2	9.04**	2.07**	9.82**
	[8.96,9.12]	[2.03,2.11]	[9.74,9.90]
2021 Q3	9.61**	2.37**	10.53**
	[9.53,9.69]	[2.33,2.41]	[10.45,10.62]
2021 Q4	10.14**	2.59**	11.21**
	[10.06,10.22]	[2.54,2.63]	[11.12,11.29]
2022 Q1	11.15**	2.98**	12.40**
	[11.07,11.23]	[2.94,3.02]	[12.31,12.49]
2022 Q2	11.58**	3.35**	13.03**
	[11.49,11.66]	[3.31,3.40]	[12.94,13.12]
2022 Q3	11.82**	3.67**	13.47**
	[11.74,11.91]	[3.62,3.71]	[13.38,13.56]
2022 Q4	12.03**	3.73**	13.80**
	[11.94,12.11]	[3.69,3.78]	[13.71,13.89]
_cons	3.13**	0.52**	3.48**
	[3.05,3.22]	[0.47,0.56]	[3.39,3.57]
N	33,039,840		

* p<0.05 ** p<0.01 based on two-tailed tests

© 2024 Shen Y et al. *JAMA Network Open.*

Appendix eTable 2b Complete results of main model—intensity of visits

Model specification: Ordinary least square model with individual fixed effects and all variables listed in the table below.

	**Intensity of visit conditional on having visit (log transformed visit volume)**		
	**MTF**	**civilian**	**Any setting**
**Military Treatment Facility core mental health provider capacity**			
adequate	0.00	0.00	0.00
	[0.00,0.00]	[0.00,0.00]	[0.00,0.00]
shortage (<1 provider per 6000 beneficiaries)	0.01*	0.01	0.01**
	[0.00,0.01]	[-0.01,0.03]	[0.00,0.01]
no MTF core provider	-0.12**	-0.01	-0.08**
	[-0.13,-0.10]	[-0.05,0.02]	[-0.09,-0.07]
**Individual time-varying characteristics**			
Married	0.01*	0.04**	0.02**
	[0.00,0.02]	[0.02,0.07]	[0.01,0.02]
Got divorced	-0.00	-0.02	-0.01
	[-0.01,0.01]	[-0.04,0.01]	[-0.02,0.00]
Got married	-0.02**	-0.03*	-0.02**
	[-0.03,-0.01]	[-0.06,-0.00]	[-0.03,-0.01]
Demoted	0.28**	0.14**	0.29**
	[0.27,0.29]	[0.09,0.19]	[0.28,0.30]
Added a dependent	-0.05**	-0.05**	-0.05**
	[-0.06,-0.04]	[-0.07,-0.03]	[-0.06,-0.05]
Promoted to senior rank	-0.01	-0.01	-0.01*
	[-0.01,0.00]	[-0.03,0.02]	[-0.02,-0.00]
Transferred to a different occupation category	0.01	0.01	-0.00
	[-0.01,0.02]	[-0.04,0.05]	[-0.02,0.01]
Moved to different community	-0.07**	-0.09**	-0.08**
	[-0.07,-0.06]	[-0.10,-0.08]	[-0.08,-0.08]
Returned to US in prior quarter	0.12**	0.06*	0.10**
	[0.10,0.13]	[0.00,0.11]	[0.09,0.12]
Return to US in current quarter	-0.26**	-0.21**	-0.28**
	[-0.27,-0.24]	[-0.28,-0.14]	[-0.29,-0.26]
Reside in rural community	-0.01**	-0.04	-0.02**
	[-0.02,-0.00]	[-0.07,0.00]	[-0.03,-0.01]
number of civilian providers per capita (log transformed)	0.07**	0.02	0.03**
	[0.06,0.08]	[-0.01,0.04]	[0.02,0.04]

© 2024 Shen Y et al. *JAMA Network Open.*

quadratic of number of civilian providers per capita (log transformed)	-0.01**	0.00	-0.00
	[-0.01,-0.01]	[-0.00,0.01]	[-0.00,0.00]
**Year-quarter indicators**			
2016 Q1	0.00	0.00	0.00
	[0.00,0.00]	[0.00,0.00]	[0.00,0.00]
2016 Q2	-0.05**	-0.05**	-0.05**
	[-0.06,-0.04]	[-0.08,-0.02]	[-0.06,-0.04]
2016 Q3	-0.07**	-0.07**	-0.07**
	[-0.08,-0.06]	[-0.11,-0.04]	[-0.08,-0.06]
2016 Q4	-0.08**	-0.10**	-0.08**
	[-0.09,-0.07]	[-0.13,-0.06]	[-0.09,-0.07]
2017 Q1	-0.01**	-0.06**	-0.01*
	[-0.03,-0.00]	[-0.10,-0.03]	[-0.02,-0.00]
2017 Q2	-0.01	-0.05**	-0.00
	[-0.02,0.00]	[-0.08,-0.01]	[-0.01,0.01]
2017 Q3	-0.03**	-0.06**	-0.02**
	[-0.04,-0.02]	[-0.10,-0.03]	[-0.03,-0.01]
2017 Q4	-0.04**	-0.07**	-0.03**
	[-0.05,-0.03]	[-0.10,-0.03]	[-0.04,-0.02]
2018 Q1	0.01	-0.03	0.02**
	[-0.00,0.02]	[-0.06,0.01]	[0.01,0.03]
2018 Q2	0.01*	-0.00	0.03**
	[0.00,0.02]	[-0.04,0.03]	[0.02,0.04]
2018 Q3	-0.01*	-0.01	0.01
	[-0.03,-0.00]	[-0.05,0.03]	[-0.00,0.02]
2018 Q4	-0.01*	-0.00	0.01
	[-0.02,-0.00]	[-0.04,0.03]	[-0.00,0.02]
2019 Q1	0.03**	0.02	0.06**
	[0.02,0.04]	[-0.01,0.06]	[0.05,0.07]
2019 Q2	0.04**	0.06**	0.08**
	[0.03,0.06]	[0.03,0.10]	[0.07,0.09]
2019 Q3	0.03**	0.08**	0.08**
	[0.02,0.05]	[0.05,0.12]	[0.07,0.09]
2019 Q4	0.04**	0.09**	0.09**
	[0.03,0.05]	[0.05,0.12]	[0.07,0.10]
2020 Q1	0.07**	0.12**	0.13**
	[0.06,0.08]	[0.08,0.15]	[0.12,0.14]
2020 Q2	0.03**	0.19**	0.10**
	[0.02,0.04]	[0.15,0.22]	[0.09,0.11]

© 2024 Shen Y et al. *JAMA Network Open.*

2020 Q3	0.05**	0.20**	0.13**
	[0.04,0.06]	[0.17,0.24]	[0.11,0.14]
2020 Q4	0.04**	0.24**	0.12**
	[0.03,0.05]	[0.20,0.28]	[0.11,0.13]
2021 Q1	0.07**	0.28**	0.15**
	[0.05,0.08]	[0.24,0.32]	[0.14,0.16]
2021 Q2	0.05**	0.30**	0.15**
	[0.04,0.06]	[0.26,0.34]	[0.14,0.16]
2021 Q3	0.02**	0.28**	0.13**
	[0.01,0.03]	[0.24,0.32]	[0.12,0.15]
2021 Q4	0.01	0.30**	0.13**
	[-0.00,0.02]	[0.26,0.34]	[0.12,0.14]
2022 Q1	0.03**	0.34**	0.17**
	[0.02,0.04]	[0.30,0.38]	[0.16,0.18]
2022 Q2	0.01*	0.33**	0.17**
	[0.00,0.02]	[0.29,0.36]	[0.16,0.18]
2022 Q3	-0.01	0.31**	0.17**
	[-0.02,0.00]	[0.27,0.35]	[0.16,0.18]
2022 Q4	-0.01*	0.32**	0.18**
	[-0.02,-0.00]	[0.28,0.36]	[0.17,0.19]
_cons	0.82**	0.83**	0.85**
	[0.80,0.83]	[0.79,0.87]	[0.83,0.86]
N	2,907,494	622,088	3,211,538

* p<0.05 ** p<0.01 based on two-tailed tests

© 2024 Shen Y et al. *JAMA Network Open.*

Appendix eTable 3 Separate coefficient estimates between pre- and post-COVID periods

	Probability of any visit			Intensity of visit conditional on having visit (log transformed visit volume)		
	**MTF**	**civilian**	**Any setting**	**MTF**	**civilian**	**Any setting**
**Pre-Covid**						
MTF adequate (reference)	0.00	0.00	0.00	0.00	0.00	0.00
Shortage (<1 provider per 6000 beneficiaries)	0.13**	-0.06**	0.11**	0.02**	0.00	0.01**
	[0.09,0.18]	[-0.09,-0.04]	[0.06,0.16]	[0.01,0.03]	[-0.02,0.03]	[0.00,0.02]
No MTF core provider	-2.21**	1.09**	-0.82**	-0.11**	-0.01	-0.10**
	[-2.30,-2.13]	[1.02,1.15]	[-0.92,-0.73]	[-0.13,-0.09]	[-0.05,0.03]	[-0.11,-0.08]
**Post-Covid (2020Q2 and onward)**						
MTF adequate (reference)	0.00	0.00	0.00	0.00	0.00	0.00
Shortage (<1 provider per 6000 beneficiaries)	-0.27**	0.45**	0.00	-0.01*	0.01	0.00
	[-0.33,-0.22]	[0.42,0.47]	[-0.06,0.06]	[-0.02,-0.00]	[-0.01,0.04]	[-0.00,0.01]
No MTF core provider	-3.09**	1.32**	-1.58**	-0.12**	-0.02	-0.05**
	[-3.19,-2.98]	[1.24,1.40]	[-1.70,-1.46]	[-0.14,-0.11]	[-0.06,0.02]	[-0.07,-0.04]
N	33,039,840			2,907,494	622,088	3,211,538

* p<0.05 ** p<0.01 based on two-tailed tests

© 2024 Shen Y et al. *JAMA Network Open.*

Appendix eTable 4 Sensitivity analysis using alternate threshold to define provider shortage (<1 provider per 9000 relevant population)

	Probability of any visit			Intensity of visit conditional on having visit (log transformed visit volume)		
	**MTF**	**civilian**	**Any setting**	**MTF**	**civilian**	**Any setting**
MTF capacity (adequate is reference group)						
Adequate	0.00	0.00	0.00	0.00	0.00	0.00
Shortage (<1 provider per 9000 beneficiaries)	-0.15**	-0.10**	-0.19**	0.01**	0.02*	0.00
	[-0.19,-0.10]	[-0.13,-0.08]	[-0.23,-0.15]	[0.00,0.01]	[0.00,0.04]	[-0.01,0.01]
No MTF core provider	-2.61**	1.04**	-1.26**	-0.12**	-0.01	-0.08**
	[-2.68,-2.54]	[0.98,1.09]	[-1.34,-1.19]	[-0.13,-0.10]	[-0.04,0.02]	[-0.09,-0.07]
N	33,039,840	33,039,840	33,039,840	2,907,494	622,088	3,211,538

* p<0.05 ** p<0.01 based on two-tailed tests

© 2024 Shen Y et al. *JAMA Network Open.*

Appendix eTable 5 Sensitivity analysis using two-dimensional fixed effects models (individual and community fixed effects)

	Probability of any visit			Intensity of visit conditional on having visit (log transformed visit volumn)		
	**MTF**	**civilian**	**Any setting**	**MTF**	**civilian**	**Any setting**
MTF capacity (adequate is reference group)						
adequate	0.00	0.00	0.00	0.00	0.00	0.00
shortage (<1 provider per 6000 beneficiaries)	-0.08*	0.34**	0.13**	-0.01**	-0.01	-0.01*
	[-0.14,-0.02]	[0.30,0.37]	[0.07,0.19]	[-0.02,-0.01]	[-0.03,0.00]	[-0.01,-0.00]
no MTF core provider	-1.14**	0.21*	-1.05**	-0.09**	-0.01	-0.05*
	[-1.47,-0.82]	[0.01,0.40]	[-1.40,-0.70]	[-0.13,-0.05]	[-0.08,0.06]	[-0.09,-0.01]
N	32,886,804			2,604,860	475,978	2,900,762

* p<0.05 ** p<0.01 based on two-tailed tests

© 2024 Shen Y et al. *JAMA Network Open.*
