# Supplementary material for: Core Mental Health Clinician Capacity and Use Rates in the US Military
Source: JAMA Netw Open. 2024 Sep 18;7(9):e2434246. doi: 10.1001/jamanetworkopen.2024.34246 (PMC11411380; doi:10.1001/jamanetworkopen.2024.34246)
Supplement: Supplement 2. — Data Sharing Statement [file jamanetwopen-e2434246-s002.pdf]

Data Sharing Statement

Shen. Core Mental Health Clinician Capacity and Use Rates in the US Military. *JAMA Netw Open*. Published online September 18, 2024. doi:10.1001/jamanetworkopen.2024.34246

Data
Data available: No

Additional Information
Explanation for why data not available: Data is proprietary and owned by the Defense Health Agency. We will provide a data dictionary upon request.
